# P340: Infectious risk management in health care facilities in guinea

**DOI:** 10.1186/2047-2994-2-S1-P340

**Published:** 2013-06-20

**Authors:** MM Diallo, S Conde, B Diallo, T Diallo, AV Dembele

**Affiliations:** 1 Focal Point RIPAQS , Ignace Deen NH, Conakry, Guinea; 2 DNEHS , MSHP, Conakry, Guinea; 3 Physician Standards and Procedures section DNEHS,Conakry, Guinea; 4 Ignace Deen NH, Conakry, Guinea

## Introduction

This study of the application of quality standards in the process of seeking care aims to contribute to improving the safety of injections, dressings and biomedical waste management in health institutions.

## Methods

This is a descriptive study on the cross-device management services offered in nursing health facilities. The technique used for data collection is that of Interview. It ran from a questionnaire on the device provides nursing care in nine health facilities targets. This is a sample having a size of 52 subjects distributed among the strata according to their size represented by the staff working in maternity services health facilities drawn.

## Results

The main results are:

i) The professional nursing is the most dominant with 34.59%, ii) Approximately 20% of respondents are on the verge of retirement, and 15% over 50 years, ii) The ability to recognize workers across their work clothes is very low, iii) There is a weakness in the procedures dispensation injections and dressings, iv) Inadequate hand washing device (38%), v) Low waste identification based bags garbage and inadequate transportation, vi) disinfection of medical devices violating procedures, vii) Low availability of clean cloth, viii) Acts and treatment protocols used infrequently and ix) The third of health professionals do not have standards prevention of infectious risk.

## Conclusion

We recommend renewing the staff of the maternity staff, train staff in the use of care protocols to improve the availability of water, garbage bags and disinfection equipment, injection materials and dressings and strengthen personal protection measures.

## Disclosure of interest

None declared

